# Supplementary material for: FXR Agonism with Bile Acid Mimetic Reduces Pre-Clinical Triple-Negative Breast Cancer Burden
Source: Cancers (Basel). 2024 Mar 30;16(7):1368. doi: 10.3390/cancers16071368 (PMC11011133; doi:10.3390/cancers16071368)
Supplement: Supplementary file 1 [file cancers-16-01368-s001.zip › Figures S1-S4 .pdf]

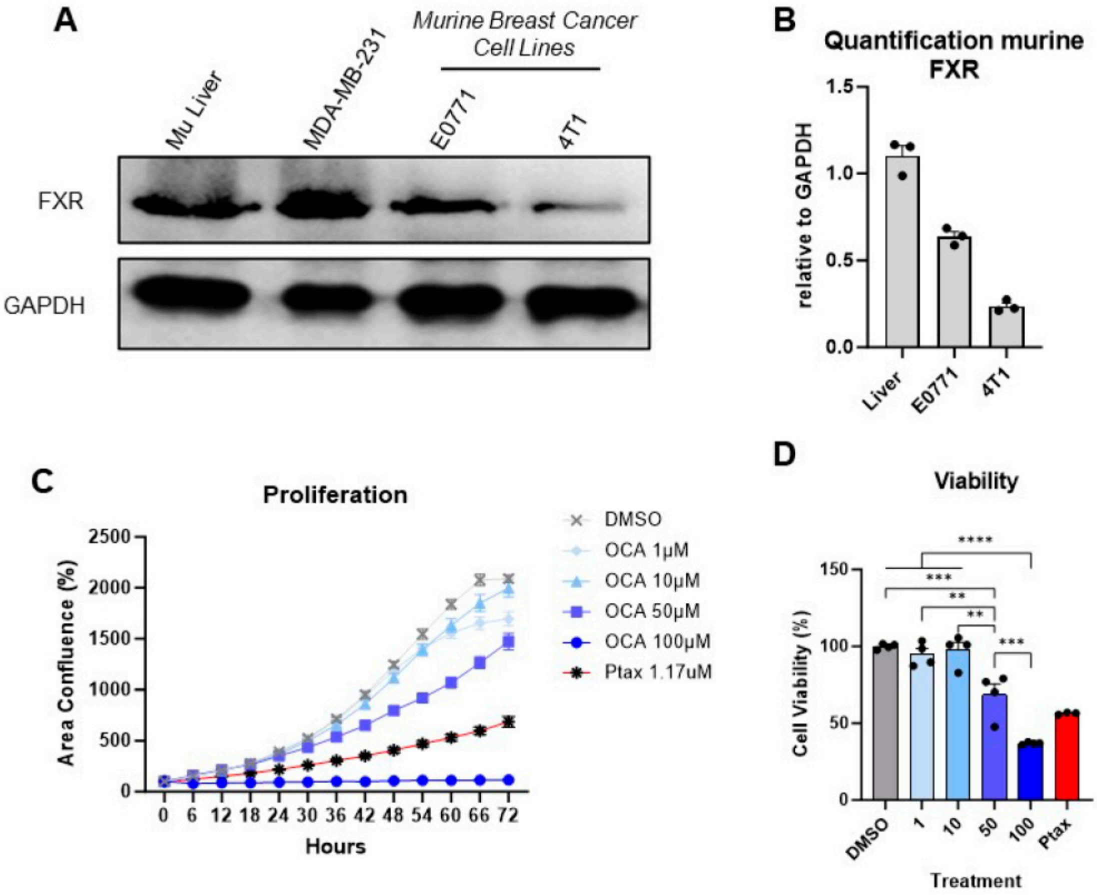

Figure S1. Impact of FXR agonism on cell proliferation, viability, and migration in murine cells. (A-B). Murine TNBC cell lines E0771 and 4T1 were immunoblotted for FXR with murine (mu) liver as a positive control and human MDA-MB-231 TNBC cells were included for comparison. GAPDH was the loading control. Representative images are shown out of N $n = 3$ Western immunoblots. (B). Im-ages were quantified using ImageJ with the relative intensity of protein expression of FXR normal-ized to GAPDH loading control with mean -/+ SEM shown. (C-D). E0771 TNBC cell lines were plated in 96 well plates at 1000 cells per well and IncuCyte Live Cell Imager quantified data at the indicated time-points. Cells were treated with increasing concentrations (0–100 μM) of obeticholic acid (“OCA”, INT-747). Paclitaxel (Ptax) was used as a positive control at 1.17 μM. C. Proliferation was measured as area confluence (%). (D). Cell viability was quantified by MTT assay at 64 h time-point. Statistical analysis was conducted using Fisher�s LSD or one-way ANOVA. Experiments include nn = 4–8 technical replicates and are representative of nN = 3–4 biological replicates. Data are shown as means −/+ SEM. **$p < 0.01$, ***$p < 0.001$, ****$p < 0.0001$; .

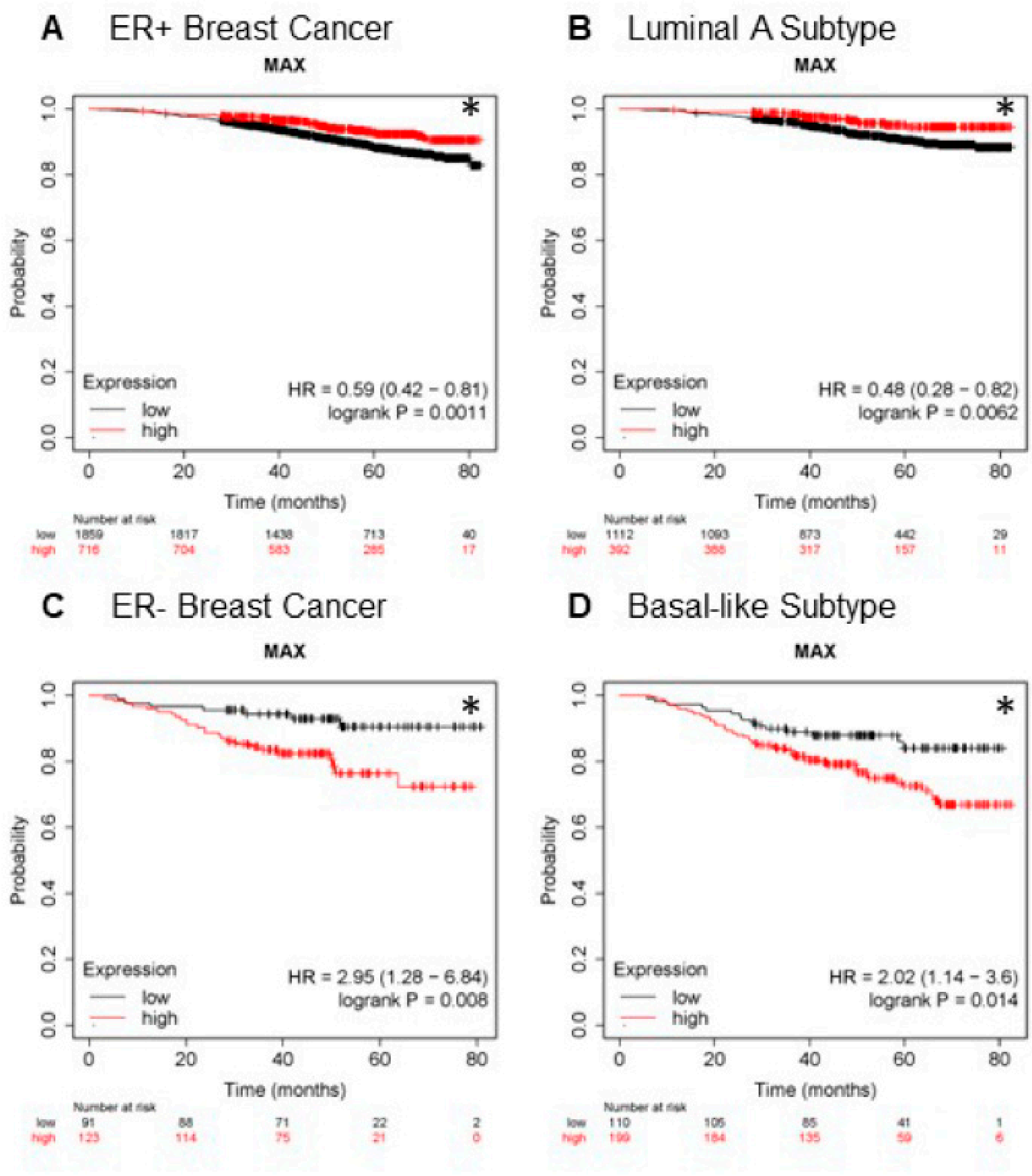

Figure S2. Higher MAX expression displays divergent overall survival in patients with ER+ com-pared to ER− breast cancer subtypes. Overall survival was analyzed in KMPlotter using RNAseq data in estrogen receptor-positive (ER+) breast cancer (A), luminal A ER+ breast cancer subtype (B), ER− breast cancer (C), and basal-like TNBC breast cancer subtypes (D). The number of patients at the indicated time in each month is shown below each graph for low and high expressors. High expression of MAX (red) is compared to low expression (black) with hazard ratios (HR) and log-rank tests that indicate p-value <0.05 as noted by an asterisk (*).

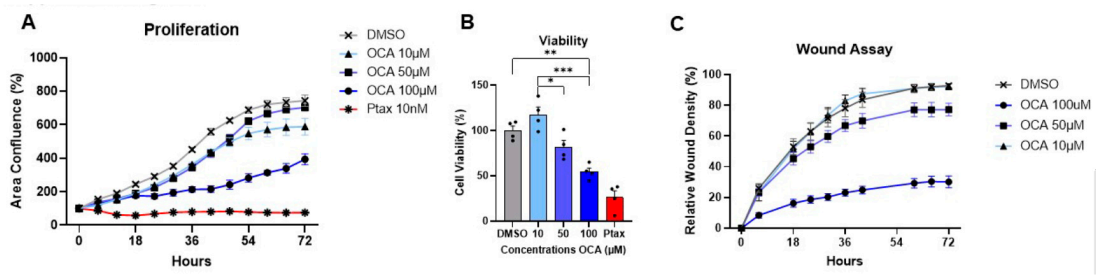

Figure S3. Impact of FXR agonism on cell proliferation, viability, and migration in SUM159 cells. (A-B). SUM159 TNBC cell lines were plated in 96 well plates at 1250 cells per well and IncuCyte Live Cell Imager

quantified data at the indicated time-points. Cells were treated with increasing concentra-tions (0–100 μM) of obeticholic acid (OCA, INT-747). Paclitaxel (Ptax) was used as a positive control at 10 nM. A. Proliferation was measured as area confluence (%). (B). Cell viability was quantified by MTT assay at 64 h time-point. (C). Cell migration was quantified using the IncuCyte by a migration wound or scratch assay. SUM159 cells were plated in 96 well plates at 7500 cells per well. Quan-tification of relative wound density (%) is reported. Statistical analysis was conducted using Fisher's LSD or one-way ANOVA. Experiments include nn = 4 technical replicates and are representative of nN = 3 biological replicates. Data are shown as means −/+ SEM. *$p < 0.05$, **$p < 0.01$, ***$p < 0.001$.

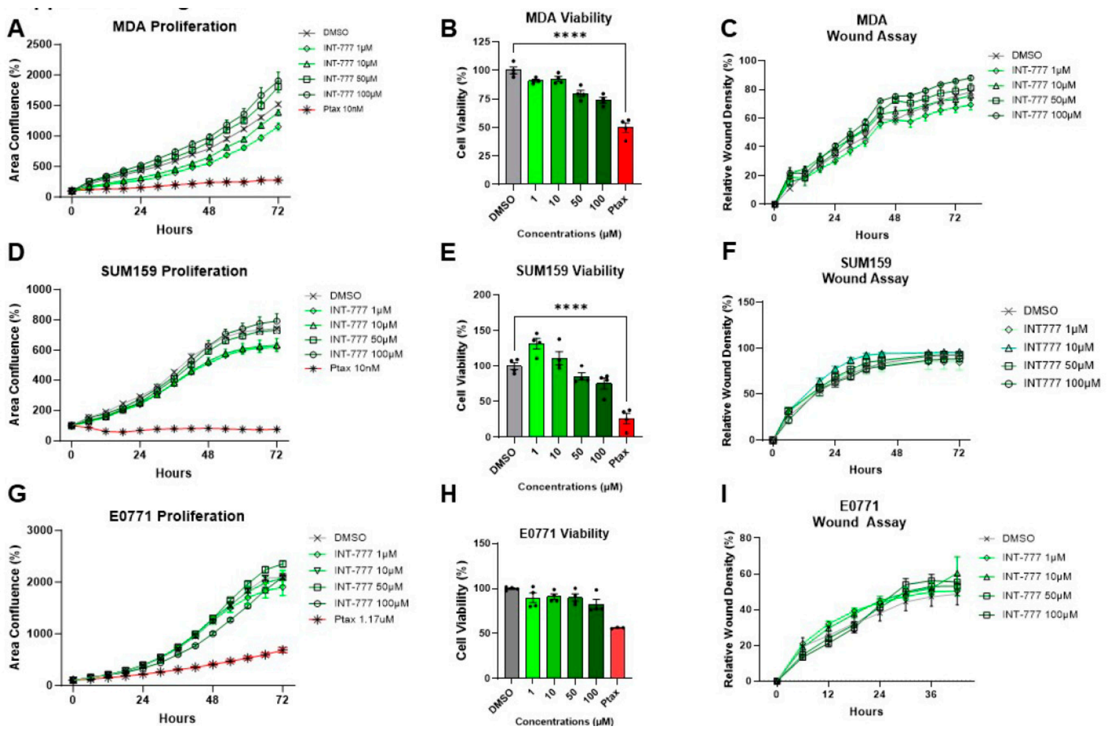

Figure S4. Impact of TGR5 agonism on cell proliferation, viability, and migration in MDA-MB-231, SUM159, and E0771 cells. A-F. MDA-MB-231 and SUM159 TNBC human cell lines were plated in 96 well plates at 1250 cells per well. G-I. Murine E0771 TNBC cell line was plated in 96 well plates at 1000 cells per well. IncuCyte Live Cell Imager quantified data at the indicated time-points. Cells were treated with increasing concentrations (0–100 μM) of TGR5 ligand INT-777. Paclitaxel (Ptax) was used as a positive control at 10 nM. A, D, G: Proliferation was measured as area confluence (%). B, E, H: Cell viability was quantified by MTT assay at 64 h time-point. Paclitaxel (Ptax) was used as a positive control at 1.17 μM. C, F, I: Cell migration was quantified using the IncuCyte by a mi-gration wound or scratch assay. MDA-MB-231, SUM159, or murine E0771 cells were plated in 96 well plates at 7500 cells per well for human lines or 3000 cells per well for murine lines. Quantifi-cation of relative wound density (%). Statistical analysis was conducted using Fisher's LSD or one-way ANOVA. Experiments include nn = 4–8 technical replicates and are representative of nN = 3–4 biological replicates. Data are shown as means −/+ SEM. ****$p < 0.0001$.
