# Supplementary material for: FXR Agonism with Bile Acid Mimetic Reduces Pre-Clinical Triple-Negative Breast Cancer Burden
Source: Cancers (Basel). 2024 Mar 30;16(7):1368. doi: 10.3390/cancers16071368 (PMC11011133; doi:10.3390/cancers16071368)
Supplement: Supplementary file 1 [file cancers-16-01368-s001.zip › Supplemental Video (SV) Files.pdf]

Supplemental Video (SV) Files: Impact of FXR agonism on proliferation. SV1 MDA cells from 0–72 h with DMSO control or 50μM OCA (SV2). MDA-MB-231 TNBC cell lines were plated in 96 well plates at 1250 cells per well and IncuCyte Live Cell Imager quantified data at the indicated time- points. Cells were treated with 0 or 50 μM of obeticholic acid (OCA, INT-747) in DMSO. Prolifer-ation was measured as area confluence (%). Representative images at time 0, 6, 36, and 72 h are shown for DMSO and 50 μM OCA in Figure 4C. Experiments include n = 4 technical replicates, with n = 3 biological replicates. Videos are representative of 1 well; Figure S1: Impact of FXR agonism on cell proliferation, viability, and migration in murine cells, Figure S2: Higher MAX expression dis-plays divergent overall survival in patients with ER+ compared to ER− breast cancer subtypes, Figure S3: Impact of FXR agonism on cell proliferation, viability, and migration in SUM159 cells,Figure S4: Impact of TGR5 agonism on cell proliferation, viability, and migration in MDA-MB-231, SUM159, and E0771 cells.
