# Supplementary material for: FXR Agonism with Bile Acid Mimetic Reduces Pre-Clinical Triple-Negative Breast Cancer Burden
Source: Cancers (Basel). 2024 Mar 30;16(7):1368. doi: 10.3390/cancers16071368 (PMC11011133; doi:10.3390/cancers16071368)
Supplement: Supplementary file 1 [file cancers-16-01368-s001.zip › Table S1.pdf]

Table S1. Antibodies and Reagents.

Antibody	Species Specificity	Host	Company	Catalog number
Anti-FXR	Human, Mouse	Polyclonal Rabbit	Invitrogen, Waltham, MA	PA5-40755
Anti-FXR/NR1H4	Mouse	Polyclonal Rabbit	Abcam, Waltham, Boston	ab235094
Anti-GAPDH (1E6D9)	Human, Mouse	Monoclonal Mouse	Proteintech, Rosemont, IL	60004-1-Ig
**Drugs**	**Alternative names**	**Target**	**Company**	**Catalog number**
INT-747	Obeticholic acid (OCA)	FXR Agonist	MedChemExpress, Monmouth, NJ	HY-12222
INT-777	S-EMCA	TGR5 Agonist	MedChemExpress, Monmouth, NJ	HY-15677
Paclitaxel	-	Chemotherapy	AstaTech Inc., Bristol, PA	N88686
Paclitaxel	-	Chemotherapy	MedChemExpress, Monmouth, NJ	HY-B0015
