# Supplementary figures and images for: FXR Agonism with Bile Acid Mimetic Reduces Pre-Clinical Triple-Negative Breast Cancer Burden
Source: Cancers (Basel). 2024 Mar 30;16(7):1368. doi: 10.3390/cancers16071368 (PMC11011133; doi:10.3390/cancers16071368)

Uncropped  
Westerns

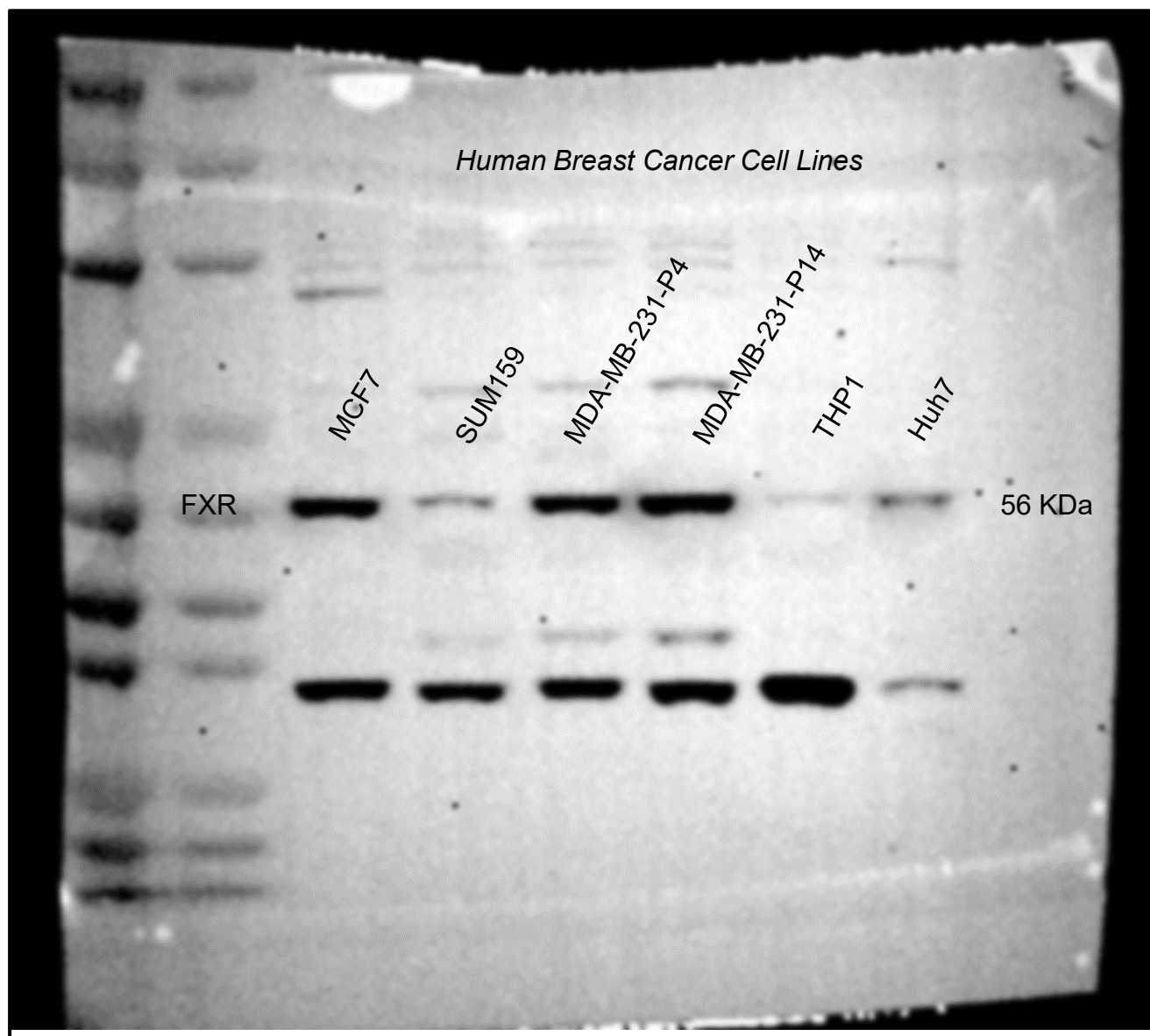

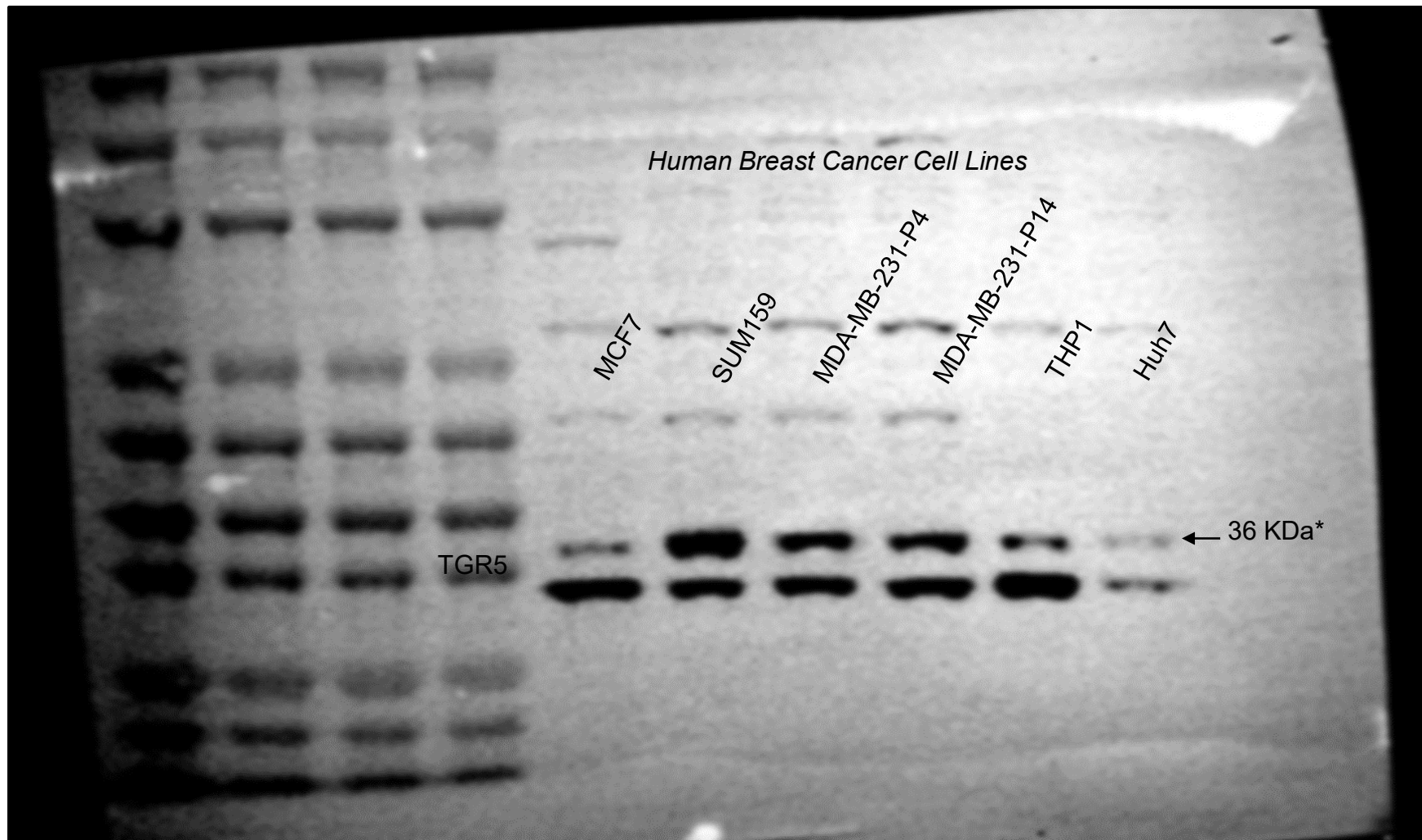

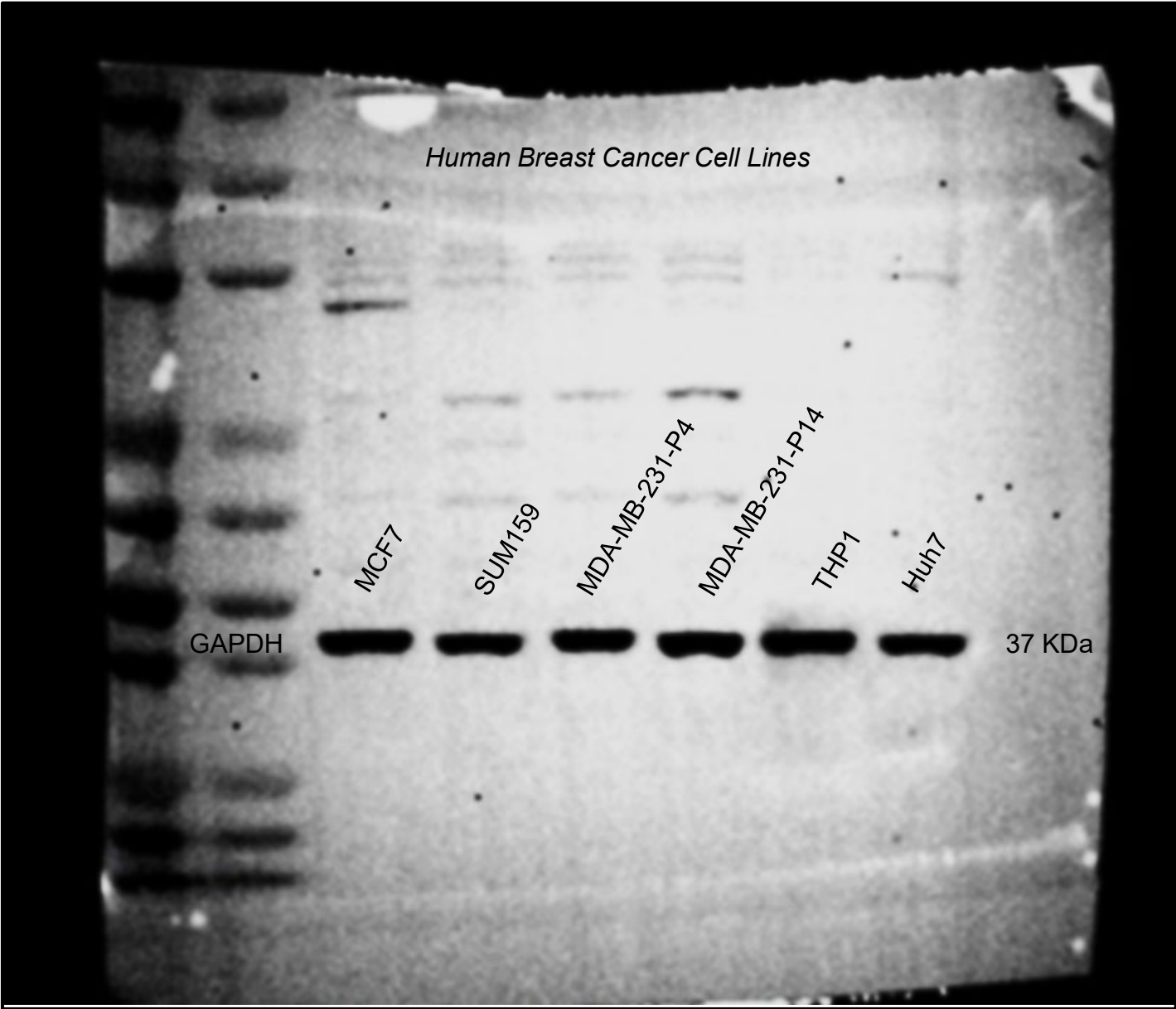

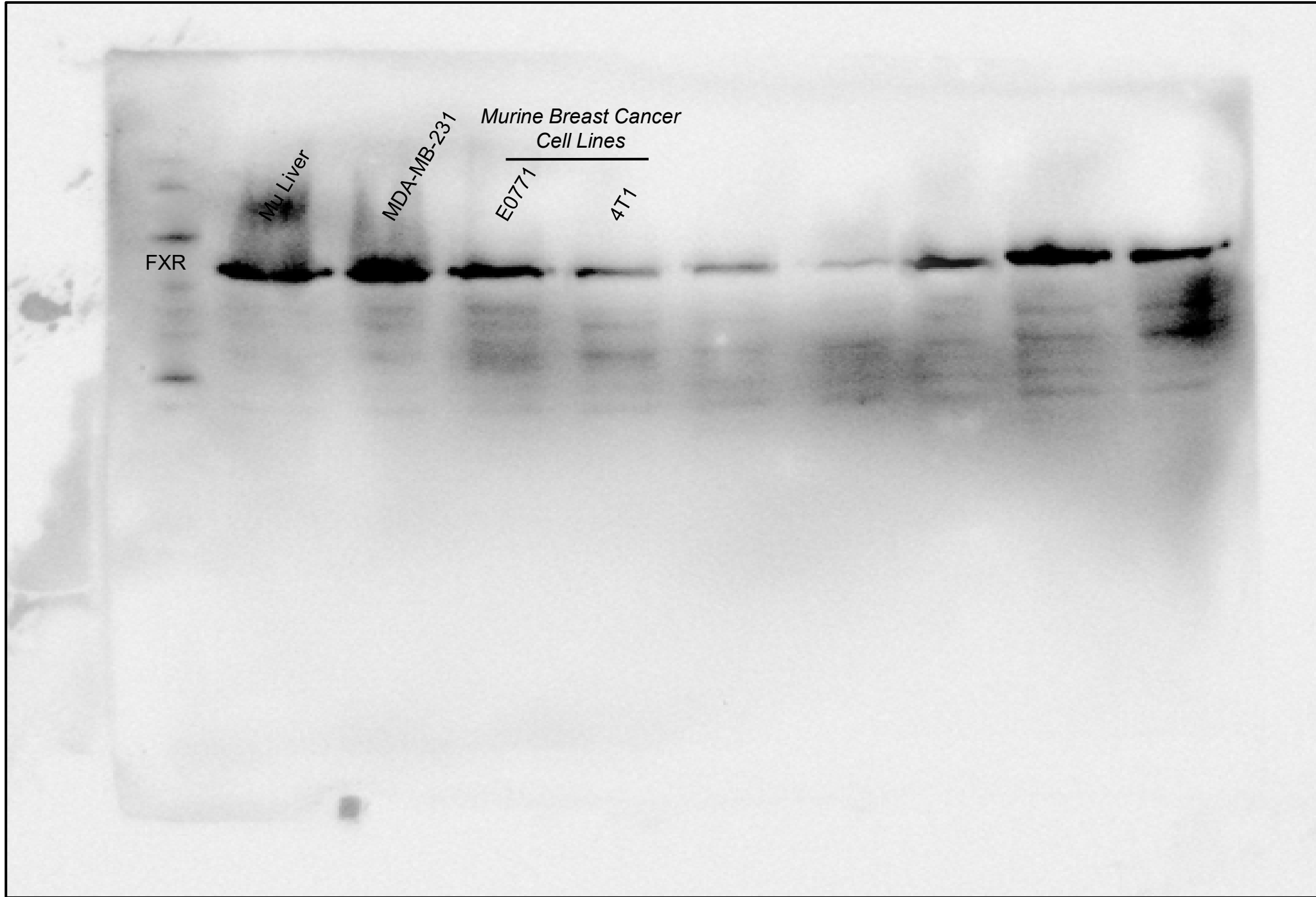

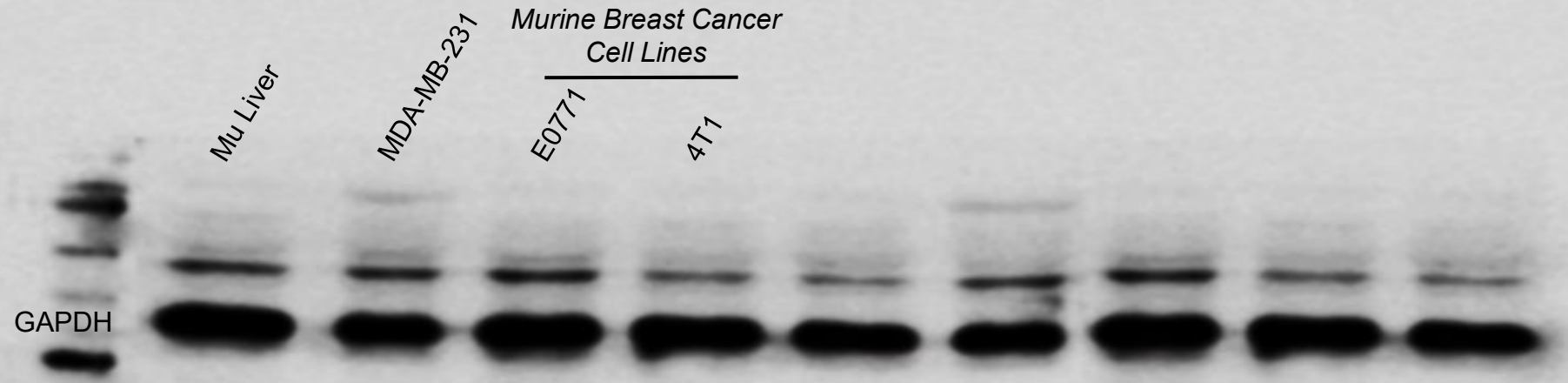

Supplement: Supplementary file 1 [file cancers-16-01368-s001.zip › cancers-2865200-original_images.pdf]
